# Enhanced Invasion of Metastatic Cancer Cells via Extracellular Matrix Interface

**DOI:** 10.1371/journal.pone.0118058

**Published:** 2015-02-23

**Authors:** Jiangrui Zhu, Long Liang, Yang Jiao, Liyu Liu, on behalf of the U.S.-China Physical Sciences-Oncology Alliance

**Affiliations:** 1 Key Laboratory of Soft Matter Physics, Institute of Physics, Chinese Academy of Sciences, Beijing, China; 2 Department of Physics, Arizona State University, Tempe, Arizona, United States of America; 3 Materials Science and Engineering, Arizona State University, Tempe, Arizona, United States of America; Seoul National University, KOREA, REPUBLIC OF

## Abstract

Cancer cell invasion is a major component of metastasis and is responsible for extensive cell diffusion into and major destruction of tissues. Cells exhibit complex invasion modes, including a variety of collective behaviors. This phenomenon results in the structural heterogeneity of the extracellular matrix (ECM) in tissues. Here, we systematically investigated the environmental heterogeneity facilitating tumor cell invasion via a combination of *in vitro* cell migration experiments and computer simulations. Specifically, we constructed an ECM microenvironment in a microfabricated biochip and successfully created a three-dimensional (3D) funnel-like matrigel interface inside. Scanning electron microscopy demonstrated that the interface was at the interior defects of the nano-scale molecular anisotropic orientation and the localized structural density variations in the matrigel. Our results, particularly the correlation of the collective migration pattern with the geometric features of the funnel-like interface, indicate that this heterogeneous *in vitro* ECM structure strongly guides and promotes aggressive cell invasion in the rigid matrigel space. A cellular automaton model was proposed based on our experimental observations, and the associated quantitative analysis indicated that cell invasion was initiated and controlled by several mechanisms, including microenvironment heterogeneity, long-range cell-cell homotype and gradient-driven directional cellular migration. Our work shows the feasibility of constructing a complex and heterogeneous *in vitro* 3D ECM microenvironment that mimics the *in vivo* environment. Moreover, our results indicate that ECM heterogeneity is essential in controlling collective cell invasive behaviors and therefore determining metastasis efficiency.

## Introduction

The most life-threatening stage of metastasis occurs when tumor cells spread from the tissue of origin and start growing in other organs. In the first critical step, called invasion, metastatic cells express metalloproteinases on their surfaces, promote basement membrane digestion and move into the surrounding extracellular matrix (ECM) [1–2]. ECM plays an important role in the process of cancer cell invasion, acting as a physical scaffold for cell movement and also as the medium of cell signal communication [3]. In tissues, cancer cells express matrix metalloproteinases (MMPs) that degrade ECM at the leading edge, generating local paths and helping the migrating cells to invade freely [4–6]. *In vivo*, invasive cell histological patterns vary from single cell invasion to collective cell invasion, even within the same tissue. Patterns range from cell acini, cords, glands, sheets, clusters and others [7–8]. Importantly, different patterns lead to different efficiencies of collective cell invasion and determine the overall severity of disease and patient survival. Therefore, it is important to understand the nature of the invasive patterns and the factors that affect them.

The collective invasive cell behaviors and patterns are greatly influenced by the heterogeneity of ECM micro-structures. At the molecular level, the ECM protein concentration and ECM components determine the ECM sub-micron structure and mechanical properties [9–14]. At the tissue scale, the structural heterogeneity and anisotropy of native tissues are primarily caused by the complex localized ECM microenvironments and their size, density and orientation. These physical characteristics, as well as the specific chemical environments consisting of oxygen, nutrition, and growth factors, vary greatly among tissues and tissue interfaces [15]. All of these ECM variations influence single cell polarity and group collective cell behaviors during invasion and lead to multiple cell invasion patterns that will ultimately shape the ECM landscape and determine the speed of metastasis.

Due to the complexity of ECM heterogeneity *in vivo*, its influence on collective cell behavior has been described but not quantified [7]. *In vitro*, studies have encountered bottlenecks due to the difficulty in constructing the ECM spatial orientation and structural defects. Hence, few modeling and theoretical analyses have been reported [6, 16]. For example, the Boyden assay is a commonly accepted method to test cancer cell invasion potential in three dimensions. However, the chamber only provides a homogeneous ECM environment, which differs from the heterogeneous tissue environment [17–18].

To further explore cell invasion, new approaches have modeled the complex microenvironment with a higher degree of similarity to the *in vivo* condition using microfluidic technology combined with optic imaging. This device offers a three-dimensional (3D) platform for cell culture and invasion that is similar to the *in vivo* microenvironment. Compared with conventional two-dimensional methods, such as scratch assays, this device provides more specificity and more accurately mimics the 3D environment for cell study [19–20]. In this manuscript, we report our recent progress on constructing a 3D matrigel-based ECM environment to study the invasive behaviors of the metastatic MDA-MB-231 breast cancer cell line. Moreover, we successfully constructed an artificial matrigel interface in 3D space. The heterogeneity of matrigel structures greatly determined the collective cell behaviors, the cell morphology and invasion efficiency. Specially, the collective cellular migration pattern was strongly coupled with the geometrical features of the funnel-like interface. Moreover, we propose a cellular automaton model [21–35] to infer the possible mechanisms that led to the observed collective invasion behavior. Our synergy of experimental and computational studies revealed that ECM heterogeneity and cell signaling, together with a chemical gradient, play essential roles in determining cancer cell invasion.

## Results

### Heterogeneous matrigel interface

**Fig 1:**
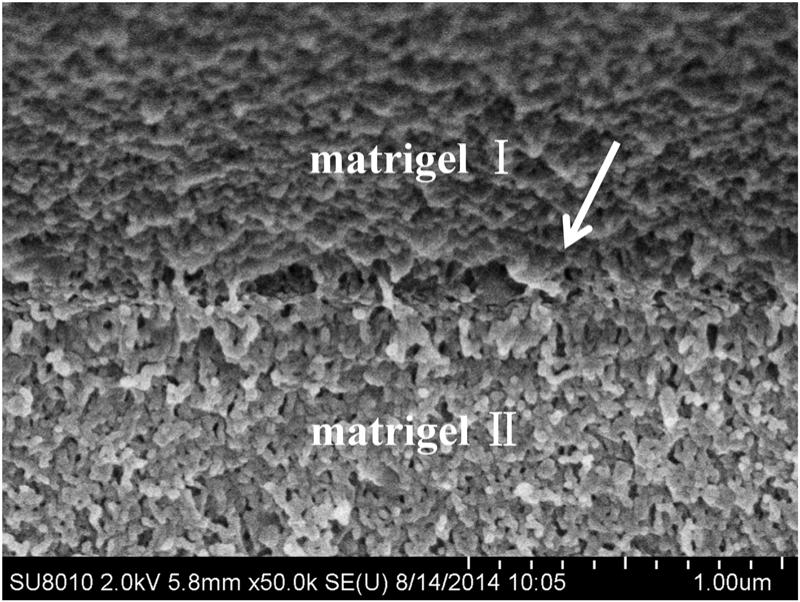
SEM image of the interface between the matrigel I and matrigel II sections. The interface has a horizontal molecular orientation and reduced localized density that produced defects inside the gel.

Matrigel is a temperature dependent gel commonly stored at 4°C. The routine procedure for preparing matrigel as *in vitro* ECM is to store the gel at 37°C. The gel then forms homogenous structures with uniform density. To create a heterogeneous matrigel structure that could simulate the non-homogeneous *in vivo* ECM microenvironment, a spatial matrigel section was prepared, cured and then joined with another matrigel section that was then cured. Two matrigel sections of identical concentration but cured at different times created an interface at their boundary. Fig. 1 is a scanning electron microscopy (SEM) image showing the details of the joint micro-scale structures. The upper section, matrigel I, was prepared and then joined with the lower section that was prepared 30 min after the upper section. Both matrigel sections had mesh structures with similar densities. However, they formed a visible vertical interface at the joint, as indicated by the white arrows. The interface had two characteristics. First, the structures had tiny cavities ranging from 100~300 nm, leading to lower localized density. Second, the molecules had horizontal polarizations along the interface, indicating that the mesh structures of the two sections do not overlap. Later experiments demonstrated and analyzed the function of this interface in determining invasive behaviors of metastatic cancer cells.

### Microfluidic setup for cell 3D invasion

**Fig 2:**
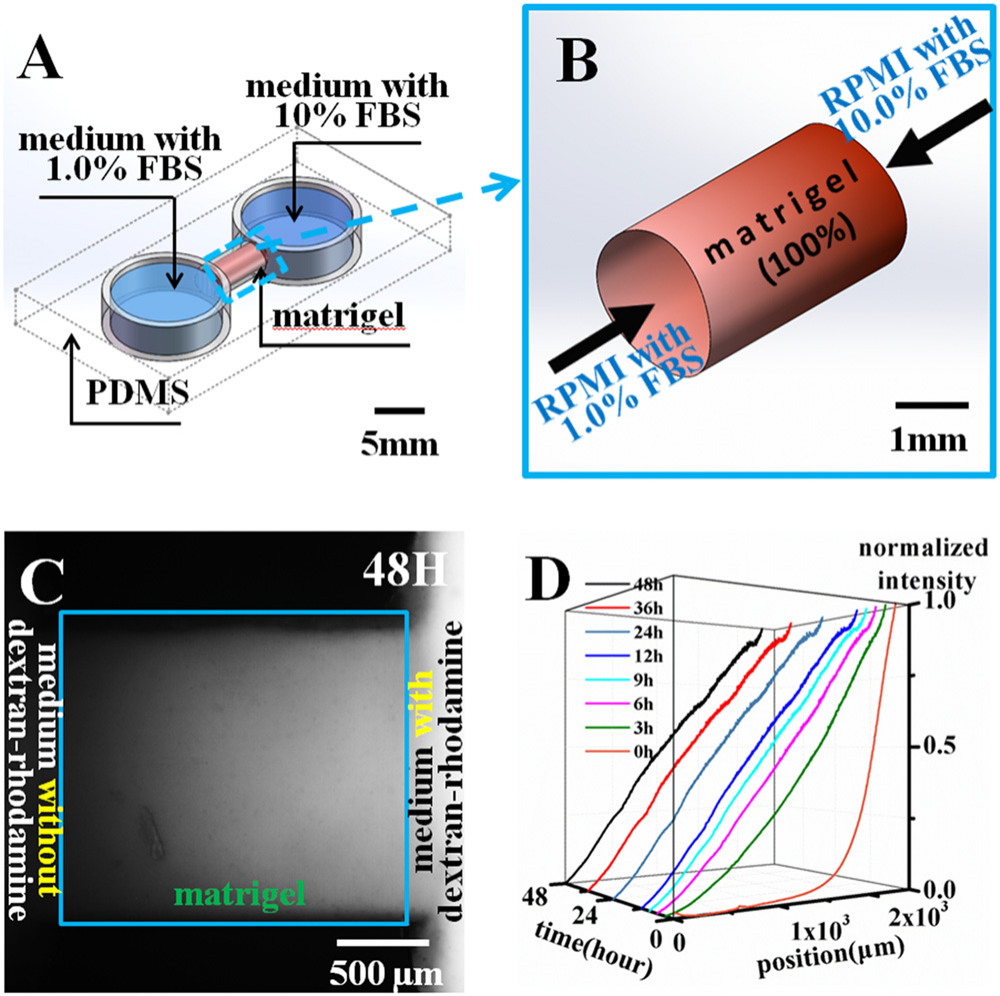
Microfluidic device and its gradient establishment in the matrigel. (A) Diagram sketch of the PDMS chip. The horizontal cylinder channel between the two chambers is filled with matrigel (red). The left chamber is filled with the medium with 1.0% FBS, whereas the right chamber is filled with the medium with 10.0% FBS. (B) The medium with 10.0% FBS and 1.0% FBS established an FBS gradient along the sandwiched matrigel. (C) Fluorescent image of the matrigel zone. The blue line indicates the matrigel. The left side with the lower gray value on shows the RPMI 1640 medium without dextran-rhodamine, and the right zone with the larger gray value represents the RPMI 1640 medium with dextran-rhodamine. The matrigel zone has a gradually decreasing gray color, indicating the establishment of a dextran-rhodamine gradient. (D) Quantitative analysis of the dextran-rhodamine gradient in the time- and space-dependent establishment in the matrigel. The gradient was established in 3 hours and remained stable after 9–48 hours.

**Fig 3:**
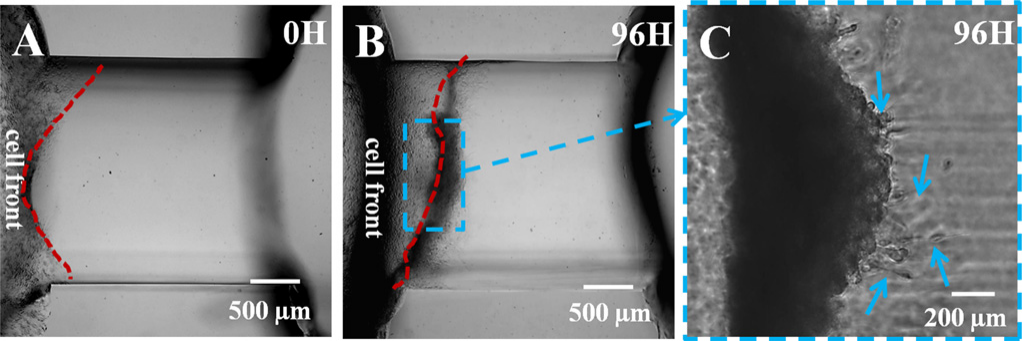
MDA-MB-231 cell invasion in the structurally homogeneous matrigel. (A) The MDA-MB-231 cells attached to the matrigel side surface wall at 0 hours. The red line shows the front of the cell group. (B) MDA-MB-231 cell invasion in the matrigel at 96 hours. The cells and matrigel digestion caused gel shrinkage compared with the cell front at 0 hours. (C) Few cells in Fig. 3B stretched out and exhibited slight invasion into the matrigel, as the white arrows denote, indicating that MDA-MB-231 cells could not invade into the stiff matrigel of 100% concentration.

To analyze how the matrigel interface influenced metastatic cell invasion in 3D space, we designed and fabricated a microfluidic chip (Fig. 2A). The dashed lines outline the cubic shape of the polydimethylsiloxane (PDMS) chip. The chip possessed two round chambers connected with a cylindrical hollow tunnel filled with cured 100% matrigel. The protein concentration was approximately 10 mg/ml, which is 3–4-fold higher than the commonly used collagen I from rat tails (3–4 mg/ml) (354236, Dow Corning, MI, U. S. A). Fetal bovine serum (FBS) is a commonly used growth factor for cancer cell growth. In cancer cell invasion *in vivo*, metastatic cells usually follow the growth factor attractant gradient. The vessel is richer in nutrition and growth factors than tissue, and these factors form a gradient that guides the direction of cell invasion. Accordingly, the experiment formed a FBS gradient that mimicked the *in vivo* microenvironment for guiding cell invasion. RPMI medium with 1.0% FBS was placed in the left chamber, and RPMI medium with 10% FBS was placed in another chamber. Fig. 2B shows that an FBS gradient developed inside the matrigel when the media with different FBS concentrations was applied at the two ends of the matrigel at 100% concentration. Metastatic MDA-MB-231 breast cancer cells were seeded onto the surface of the matrigel in the chamber containing the medium with 1.0% FBS. Each chamber was filled with 180 μl of medium to ensure even pressure differences between the two chambers. For the first 24 hours, the cell chamber was filled with 10% FBS medium to ensure normal growth of the MDA-MB-231 cells. After 24 hours, the medium was changed to 1.0% FBS medium, and the FBS gradient was established. To test the establishment and stability of the gradient, fluorescent dextran-rhodamine (70 kDa molecular weight protein, Invitrogen) at a concentration of 0.025 mg/ml was used to simulate the establishment of the FBS gradient [36–37]. The molecular weight of dextran-rhodamine is similar to bovine serum albumin at 66.5 kDa. An inverted microscope (Ti-E, Nikon) with EMCCD (Flash 4.0, Hamamatsu) was employed to capture the fluorescent signals in the matrigel. The chip with matrigel inside was placed inside a customized on-stage live cell incubator at 37.0°C, 5.0% CO_2_ and 80.0% humidity, a normal cell incubation environment. The Micro-manager software controlled the microscope to acquire time-lapse images of the fluorescent dextran-rhodamine distributions inside the matrigel every 10 min for 48 hours. Fig. 3C shows the dextran-rhodamine distribution 48 hours after the gradient was established. A visible gradient was established in the gel. This time-lapse imaging was later quantitatively analyzed by ImageJ software. The averaged gray values were obtained and analyzed from the images, which represent the dextran-rhodamine fluorescence intensities at specific locations. These values were then normalized and plotted with locations and time into the graph by Origin software, as is shown in Fig. 3D. A series of dextran-rhodamine diffusion images were taken at 10 min intervals. Each image was normalized with the maximum and minimum obtained from each image in the series [38]. The gradient profiles were analyzed by averaging a horizontal rectangle along the gel region and plotted against their position in the channel. Fig. 2D indicates that the simulated FBS gradient was established within 3 hours and maintained for up to 48 hours. The gradient test indicated that matrigel could establish a linear and stable gradient. Additionally, when medium with or without FBS was applied simultaneously to the two sides, an effective gradient formed in the matrigel zone.

### Invasion behavior of MDA-MB-231 cells in a homogeneous ECM environment

Next, the influence of the heterogeneous matrigel interface on collective cell invasion was analyzed. For comparison, the control experiment analyzed cell invasion in a homogenous ECM microenvironment. Fig. 3A shows the connecting cylindered channel filled with homogenous matrigel at 100% concentration. At 0 hours, MDA-MB-231 cells attached uniformly to the left side of the matrigel. Both chambers were filled with 10% FBS medium, which ensured cell attachment and normal growth. After 24 hours, the medium in the left chamber was replaced with medium containing 1.0% FBS. In Fig. 3B, the black shadow along the matrigel surface indicates that the cell number increased significantly due to rapid cell proliferation. After 96 hours, the matrigel was deformed, and the MDA-MB-231 cell front (red lines) moved forward to the right as they stuck to the matrigel surface. The matrigel deformation was caused by the MMPs secreted by the MDA-MB-231 cells, which degrade the matrigel [39], and the force of the interaction between the MDA-MB-231 cells and matrigel microstructure [40–42]. The former represents a chemical action, whereas the latter is a physical process. Although the convergent forces were significant and caused matrigel deformation, the cells did not obviously collectively invade the matrigel 3D structures. Fig. 3C shows the enlarged section of the cell front in Fig. 3B. The picture shows the cell invasion in 3D matrigel 520 μm over the bottom of the channel. The blue arrows indicate that the MDA-MB-231 cells only slightly invaded the matrigel. As shown, only a few cells at the front formed elliptical shapes in the matrigel, representing their slight invasions. Most of the cells behind these cells showed compact growth. Overall, the MDA-MB-231 cells could not invade the matrigel, likely due to the stiffness of the 100% matrigel. This control experiment confirmed that the cells could only exhibit intensive growth on the gel surface, without 3D invasive behavior, if the rigid gel had a uniform density.

### Constructing a heterogeneous ECM microenvironment with a matrigel interface

**Fig 4:**
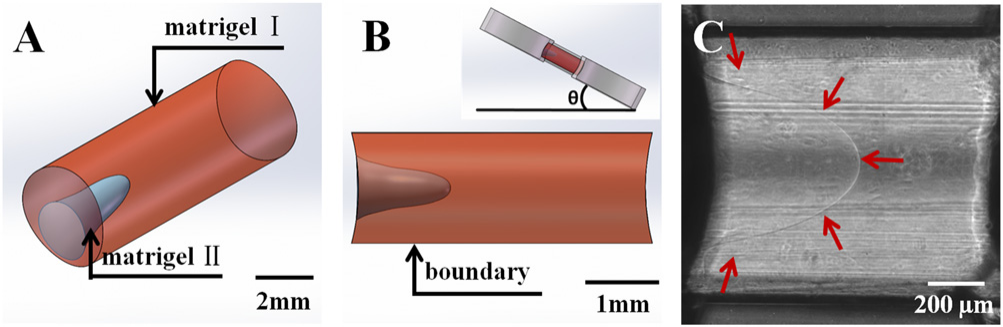
Formation of the matrigel interface. (A) Cartoon showing that the heterogeneous matrigel is composed of matrigel I (red) and matrigel II (blue). The two sections form a funnel-like 3D interface. (B) The upper inset shows the matrigel gel structure preparation. After matrigel I in the channel gelled for 30 min, the chip was tilted 30°-45°. The matrigel I formed a funnel structure due to gravity and gel adhesion. Then, matrigel II was injected into the cavity. The lower inset shows the interface from the side view. (C) The red arrows indicate the gel interface in the experiment.

Previous control experiment demonstrated that metastatic MDA-MB-231 cells were barely able to invade the ECM-like matrigel because of its stiffness. To further study the collective cell invasive behaviors in ECM with a heterogeneous microenvironment, we generated a spatial matrigel joint structure in the gel channel. Fig. 4A shows that the cylindered matrigel channel was filled with two sections of the gels: matrigel I (red) and matrigel II (blue). Specifically, matrigel II was inserted into matrigel I and formed a funnel-like 3D structure. During preparation, the cylindrical channel was first injected with 8 μm of 100% matrigel. Then, the chip was tilted 30°~45° degrees to the level platform for 30 min while the gel cured (Fig. 4B). As the matrigel had not solidified during this time and the soft matrigel adhered strongly to the PDMS channel, gravity drove the gel to the right end, creating a funnel-like structure until fully solidified. At this point, gravity combined with gel adhesion played an important role in the formation of the 3D morphology of the matrigel I. Finally, another 3 μl of 100% matrigel II was added to the cavity. The chip was then placed inside a level incubator for complete gelling. Thus, matrigel I and II combined and formed a funnel-like interface, as shown in Fig. 4B. These two sections had identical gel density, but the interface had structural differences. As shown by SEM in Fig. 1, the local density and gel molecule orientation varied from the uniform sections. The physical properties of the interface were therefore different from the homogeneous matrigel. The variations also led to changes in the optic properties, including the reflection index. Figure C shows the bright-field picture imaged by the inverted microscope. The funnel-like 3D interface is clearly visible in the channel, as indicated by the red arrows.

### Matrigel interface-guided collective MDA-MB-231 cell invasion

**Fig 5:**
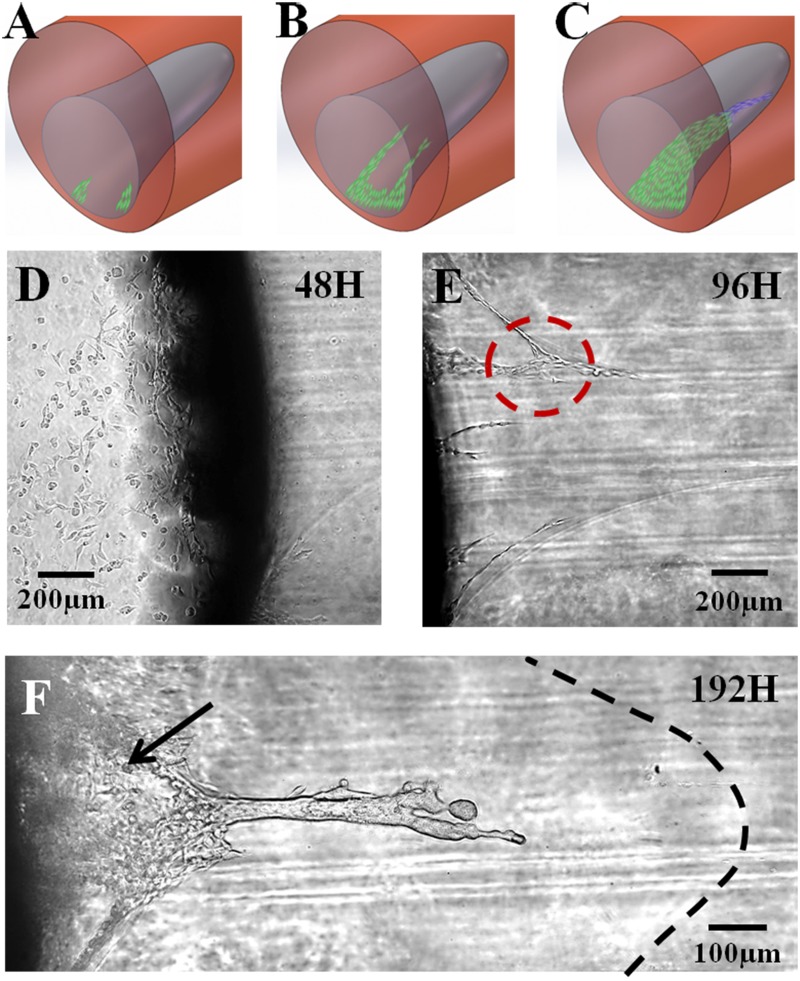
MDA-MB-231 collective cell invasion in the matrigel with 3D interface. (A-C) Cartoons explaining the different phases of collective cell invasion. (D) shows that the MDA-MB-231 cells sensed the interface and began invading the matrigel at 48 hours; (B) The MDA-MB-231 cells’ invasive branches increased following the matrigel interface at 96 hours. Some branches link and form a network, as indicated by the red circle. (C) After the partial interface was filled with the cells, the frontier cells escaped from the interface confinement and produced finger-like invasions in the homogenous matrigel, confirming the strong invasion of the MDA-MB-231 cells in heterogeneous gel space.

MDA-MB-231 cells were loaded on the left side of the heterogeneous matrigel with interfaces and cultured for 48 hours (Fig. 5D). The procedure was exactly the same as that for the control experiment shown in Fig. 3. In Fig. 5D, the shadow is the boundary of the matrigel channel and round pool and is caused by the light projection of the PDMS wall. The focal plane of the picture is on the chamber substrate. The shadow on the left is the medium chamber where MDA-MB-231 cells were previously loaded and had settled down and proliferated on the substrate. The right side is the matrigel zone, where cells attached to the left wall. Most cells on the vertical matrigel surface remained in the shadowed area and are not visible in the picture. The lower-right color of the picture indicates that after MDA-MB-231 cells had attached to the matrigel side for 48 hours, a few cells stretched out and started invading the inner matrigel strictly following the interface. The cells that had no contact with the interface in space did not invade, similar to the control experiment. These results indicate that the cells had mechanotransduction response to the structure and could sense and follow the interface. The interface plays an important role in guiding cell invasion, which could not occur in homogenous stiff matrigel. As the SEM shows, the ECM fibrils had polarizations and defects in the interface that possibly guided cell directional invasion. Besides, the heterogeneous physical constrains and tensions could induce anchor receptor redistribution in cell membrane. Thus the resulted mechanical stretching of the membranes may control pseudopodia extensions and motivate cell invasion along the interface. To clarify this process, the above schematic explains the cell invasion following the spatial matrigel interface.

After 96 hours, the cell invasion became more apparent, as shown in Fig. 5E. More MDA-MB-231 cells, forming individual branches, started invading the matrigel along the interface, both from the side and bottom. The cells invaded along the side interface, revealing that invasion still strictly followed the interface at fast speeds. In addition to the interface influence, the cell signal also played a vital role in accelerating cell invasion. Cell communication helped the 3D invading cells form a network and connect with other invading branches, as indicated by the circle outlined with red dashed lines. This connection helped the cells form a large group and invade the gel space collectively. Simultaneously, the FBS gradient also contributed and led cells to invade towards the right side [43–44].

With the benefit of interface guidance, MDA-MB-231 cells quickly invaded the matrigel. At 192 hours, the MDA-MB-231 cells presented strong collective invasive behaviors and invasion patterns in the gel. Fig. 5F shows that the cell invasion group formed in two sections of the matrigel. Early-invading cells that formed invading branches along the interface continued to invade, proliferating and merging until they connected and formed a cell plane 350 μm in length and 100 μm in width, as denoted by the black arrow. This cell plane was confined by the interface panel. Then, a dense cluster of cells emerged at its front and continued to develop into an elongated shape that invaded into the ECM ahead. We refer to this elongated cell cluster as a cell “finger”. The finger invaded horizontally toward the right of the gel, indicating that the cells had escaped from the confinement of the matrigel interface and invaded into the matrigel I section. In other words, the cells no longer invaded the gel along the interface but directly into the homogenous matrigel environment. The black dashed line represents the front of the funnel interface and shows the cell finger generated before the cells filled the interface. The boundary of the cells in the finger is not visible, indicating that the cell group had very compact structures with strong cell-cell junctions. Compared with the cell invasion behavior in the control experiment, in which only a few cells displayed slight and short-distance invasion at the cell group front, the massive finger invasion implies that the MDA-MB-231 cells had strong signal communications with each other that dominated their collective behaviors, which may explain why the cell group here could invade 100% matrigel space, unlike the control group in the matrigel with the same density. In addition, the FBS gradient contributed greatly and directed the finger invasion to the right [45].

## Discussion

Generally, the invasion process of metastatic MDA-MB-231 cells in matrigel with a funnel-like 3D interface could be considered to have three phases. In the 1^st^ phase, the cells invaded the matrigel in a few streams, primarily guided by the curved interface surface (48 hours). In the 2^nd^ phase, more cell streams appeared at the interface. At this stage, cell signaling dominated, allowing invaded streams to communicate with others and form cell networks. During the 3^rd^ phase (192 hours), the cell network turned into a continuously invading cell group that generated the finger shape and collectively invaded at the front. Simultaneously, the FBS chemical gradient and cell signaling were the dominant factors directing cell invasion to the right in the rigid and homogenous matrigel space.

To theoretically analyze the collective cell invasion in a heterogeneous matrigel microenvironment, we proposed the following mechanisms that could induce the salient features of the 3-phase invasion behavior observed here: (i) micro-environment heterogeneity; (ii) long-range homotypic attraction through cell-cell communication (primarily chemical in nature); and (iii) gradient-driven directional cellular migration. To further verify these mechanisms and obtain a deeper understanding of how they are coupled, we devised a cellular automaton (CA) model [22–35] that incorporated the aforementioned mechanisms to *qualitatively* simulate the collective cellular migration in a heterogeneous environment. We note that although the actual system is in three dimensions, we employed a two-dimensional (2D) model. This is because our previous studies have shown that 2D CA models are sufficient to qualitatively reproduce the collective cellular dynamics in invasive solid tumors [46–48] and dormant malignant tumors [49].

**Fig 6:**
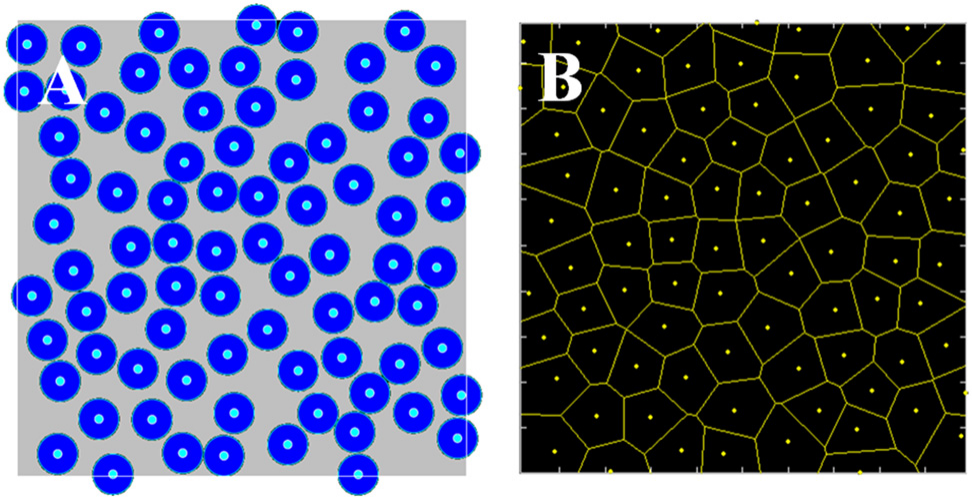
Left panel: packing of hard disks generated by random sequential addition. Right panel: associated Voronoi tessellation of the plane into polygons.

Following previous studies [46–49], a rectangular simulation domain (with an aspect ratio of 2) was divided into discrete polygons using Voronoi tessellation associated with a disordered packing of circular disks (Fig. 6). The packing was generated via random sequential addition of 80,000 hard circular disks [46]. The linear size of the simulation domain was 4000 μm x 2000 μm. Thus, the average linear size of each Voronoi polygon was approximately 10 μm, consistent with the typical size of a cell. In our simulation, a Voronoi polygon can be occupied by ECM macromolecules, a cell, or a void space. Each ECM associated Voronoi polygon possessed an effective ECM density value *ρ*, and for a void-space polygon, *ρ = 0*. We note that this density value is a simulation parameter that is proportional to but generally different from the actual ECM density (i.e., number/mass of ECM macromolecules per unit volume/area).

To mimic the heterogeneous micro-environment observed in the experiments, we considered two ECM regions with different densities, i.e., a soft region with *ρ*_*b*_ and a hard region with *ρ*_*h*_ We used a Gaussian curve to approximate the border of the two regions. The Voronoi polygons at the interface of two ECM regions had a much lower density *ρ*_*b*_ than the bulk ECM regions due to possible defects at the interface. The density values are provided in Table 1. In addition, a uniform chemical gradient applied along the horizontal direction was implicitly incorporated in our model, which biased the migration of the cells along the horizontal direction (as detailed below). We note that in general, a gradient prolife is much more complex than a uniform one. However, this approximation was sufficient for our qualitative study.

**Table 1:** Summary of the simulation parameters.

Parameter	Physical Interpretation	Value
*P*_*m*_	Migration probability of a cell	-
*P*_*0*_	Base migration probability of a cell in homogeneous ECM	0.23
*P*_*s*_	Effective ECM density for soft matrigel	1.2
*P*_*h*_	Effective ECM density for hard matrigel	1.6
*P*_*b*_	Effective ECM density for the border region	0.86
∇c	Chemical gradient	-
*R*_*c*_	Influence circle radius for homotype attraction	250 μm
r_ij_	Displacement vector from polygon *i* to polygon *j*	-
r_*ic*_	Displacement vector from polygon *i* to attraction center *c*	-
*a*_1_	Normalization parameter for the effect of ECM density	1.03
*a*_2_	Normalization parameter for the effect of homotype attraction	0.14 μm^-2^
*a*_3_	Normalization parameter for the effect of chemical gradient	0.037 μm^-2^

The parameters values are also provided whenever possible, depending on the specified parameter values and the positions of the cells, and generally vary during the simulation.

In the beginning of the simulation, cells are released and can enter the simulation domain from the left side of the domain. Simulation time is then discretized into hours. When entering the simulation domain, a cell occupies a Voronoi polygon and degrades the ECM macromolecules originally in the polygon. At each time step, every cell is checked for migration. When a cell migrates, it jumps from the current Voronoi polygon to a neighboring Voronoi polygon with a probability *P*_*m*,_ which is a key simulation parameter that depends on the ECM density of the neighboring polygon, the direction of chemical gradient and the positions of other cells. Specifically, the following CA rules are employed to determine *P*_*m*_:

1. *ECM density*: Following previous studies [46–49], we assumed that it is easier for cells to migrate in soft ECM than hard ECM. Thus, we consider the probability that a cell jumps into a Voronoi polygon *j* with *ρ*_*j*_ to be proportional to *e* ^*-ρj*^, i.e., *P*_*m*_ ∼ e^-*ρj*^
2. *Chemical gradient*: We considered that the migration direction of a cell is biased by the chemical gradient. This rule was implemented by imposing a higher migration probability if the migration direction was more aligned with the gradient direction. Suppose a cell originally occupies a Voronoi polygon *i*, the probability that the cell jumps to a neighboring Voronoi polygon j is then proportional to e^(*r*_*ij*_∇*c*)^, i.e., P_*m*_ *∼* e^(*r*_*ij*_∇*c*)^, where *r*_*ij*_ denotes the displacement vector pointing from polygon *i* to *j* and ∇*c* is the applied chemical gradient, which is selected to be the unit vector along the horizontal direction.
3. *Homotype attraction*: We consider that the direction of migration is also affected by other cells through cell-cell communication that could be chemical in nature. Without getting into the details of the cell-cell communication involved in the observed collective behavior, which requires comprehensive sub-cellular level studies, we only implemented the overall effect of such communication in our model, i.e., homotype attraction. This attraction implies that cells migrate towards one another and was implemented as follows: suppose a cell originally occupies a Voronoi polygon *i*, we construct a circle of radius *R*_c_ centered at polygon *i*, where *R*_c_ is the effective range of cell-cell communication. Then, the positions of all of the cells within the influence circle are averaged, giving the position of an effective center of attraction. Let the vector r_*ic*_ denote the displacement from polygon *i* to the averaged cell position (i.e., center of attraction). The probability that the cell jumps to a neighboring Voronoi polygon *j* is considered proportional to e^(*r*_*ij*_∙*r*_*ic*_)^, i.e.,*P*_*m*_ ∼ *e* ^(*r*_*ij*_∙*r*_*ic*_)^, where r_*ij*_ denotes the displacement vector pointing from polygon *i* to *j*.

Combining the aforementioned effects (1) to (3), the overall migration probability that a cell in polygon *i* will jump to polygon *j* is then given by the following equation:(1)

$$P_{m}\;=\;P_{0}\;\exp\;(-a_{1}\;*\;\rho_{j}\;+\;a_{2}\;*\;r_{ij}\;\cdot \;\nabla_{C}\;+\;a_{3}\;*\;r_{ij}\;\cdot \;r_{ic})$$ (1)

where *a*_1_, *a*_2_, and *a*_3_ are the normalization parameters that measure the “strength” of each effect and *P*_*0*_ is a base migration probability related to the migration velocity of a cell in homogeneous ECM. All simulation parameters were determined by comparing and matching the simulated migration patterns with the experimental observations. The values are provided in Table 1. We emphasize that the model parameters are not directly measurable from experiments at this stage, and the purpose of the model was to test the proposed mechanisms by *qualitatively* reproducing the salient features of the collective migration behavior, not to make quantitative predictions.

**Fig 7:**
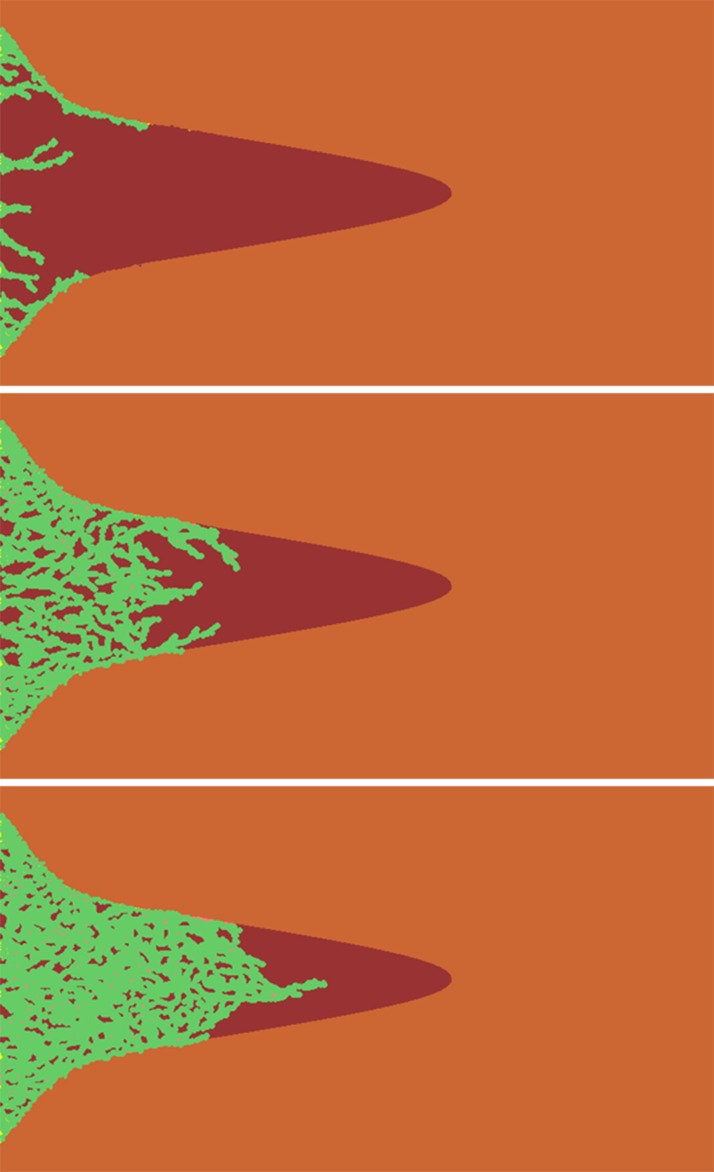
Snapshots of the simulated collective migration pattern corresponding to the three stages in the experimental observations.

The aforementioned CA rules were employed to update the position of each cell in the simulation domain, and the simulated collective migration proceeds by advancing the discrete simulation time. Fig. 7 shows the simulated migration patterns corresponding to the experimental observations shown in Fig. 5. For purposes of visualization, the following coloring scheme was used: the soft ECM region is shown in dark red, and the hard region is shown in light red (consistent with Fig. 4). The Voronoi polygons that were occupied and are being occupied by a cell are shown in blue, which reveals the migration pattern of the cells. Comparison with Fig. 5 shows that the salient features of the collective migration pattern were qualitatively reproduced. Specifically, during the first phase, the cells utilize the weak border regions due to their low density and primarily migrate within such regions. In phase 2, driven by chemical gradients and homotype attraction, cells begin to migrate within the soft ECM. As the number of cells in this region increases, the resulting strong homotype attraction “drags” the threads of cells along the borders into the soft ECM region. Finally, in phase 3, when different threads merge in the center of the soft ECM region due to the symmetry of the pattern and the chemical gradient, a dendritic branch emerges. The qualitative agreement between our simulation and experimental results suggests that the three proposed mechanisms indeed play a key role in determining the migration dynamics of the cells.

## Materials and Methods

### Matrigel SEM preparation

First, 20 μl of matrigel was dripped onto a glass slide and placed in an incubator for 30 min. Another 20 μl of matrigel was then placed next to the previous matrigel and incubated for 30 min. Then, the glass with the matrigel above was placed into a solution of 2.5% glutaraldehyde (Electron Microscopy Sciences) and 2.0% paraformaldehyde (Electron Microscopy Sciences) mixed in 0.1 M Na Cacodylate buffer (pH 7.4) overnight. After fixation, the matrigel was rinsed by the same buffer for 1 hour and immersed in a 50%, 70%, 90%, 95% and 100% ethanol series for 15 min each. Finally, the sample was dried in a critical point drier (Samdri-PVT-3D, Tousimis) and sputter coated with platinum for SEM visualization [50–51]. All images were captured by a field emission scanning electron microscope (FESEM) (SU8010, Hitachi).

### Microchip design and fabrication

To fabricate the microchip, a stainless steel tube (approximately 2 mm in diameter and 20 mm in length) was placed on the bottom center of a 60 mm petri dish. Then, PDMS (Dow Corning, USA) base and curing agent were mixed at a ratio of 10:1 in weight, and 10 ml of the mixture was prepared. The PDMS was poured into the petri dish, forming a 3.5 mm thick layer. After the air bubbles had been removed in a vacuum oven, the petri dish was placed in an 80°C hot air oven (FD 115, Binder, Ger) for 60 min until the PDMS was fully cured. Using a razor blade, the stainless steel tube was carefully peeled off the cured PDMS, and a PDMS layer with a 20 mm long channel remained. In this channel, two adjacent holes 8 mm in diameter and 3 mm apart were punched through the layer, which led to PDMS structures with a 3 mm long channel connected with vertically through two holes, as shown in Fig. 2A. This layer, together with another pre-cured 1 mm-thick PDMS layer, was plasma treated in a plasma cleaner (PDC-002, Harrick, USA) for 2 min in 29.5 W. These two layers were immediately bound together after the treatment, generating the microchip device.

### Cell culture

MDA-MB-231 cells were obtained from the American Type Culture Collection (ATCC) and were grown according to the instructions provided by the ATCC. In details, the cells were cultured in RPMI 1640 (11875–093, Invitrogen) with 10% FBS (Corning-Cellgro) and 1.0% penicillin (1.0×10^4^ units/ml) and streptomycin (10 mg/ml). Cells were seeded and grown in an incubator at 37.0°C, 5.0% CO^2^ and 80.0% humidity. Before loading into the microchips, MDA-MB-231 cells were grown in flasks in a monolayer to 80% confluence and then harvested with 0.25% trypsin-EDTA (Mediatech). These cell suspensions were then centrifuged (Heraeus multifuge X1R, Thermo, GER) at 400 x g for 5 minutes. The centrifuged cells were pelleted and re-suspended in RPMI 1640 before being loaded into the microdevice. Each cell chamber was loaded with approximately 10^4^ cells.

### Matrigel preparation and cell loading

The matrigel (356237, BD science) was stored at-20°C before usage and thawed by submerging the vial in ice at 4°C overnight. To ensure matrigel homogeneity, pipettes were all pre-cooled in the 4°C refrigerator. During the procedure, the matrigel vial was placed on an ice pack. Then, 12 μl of matrigel was obtained and injected into the chip gel channel, as shown in Fig. 2A, before the chip was placed in the incubator for 1 hour in the control experiment group or 30 min in the matrigel interface group. In the control group, the matrigel was then cured. The cell suspension with 10^4^ cells in RPMI 1640 was added to the left chamber, and pure RPMI 1640 was injected into the other chamber. The chip was thereafter tilted at 30°-45° to the level for 15–20 min at room temperature. The cell chamber would be spatially higher than the non-cell chamber and hence help the cells sediment and attach to the vertical walls of the matrigel. Then, the chip was put in the incubator for long-term incubation and analysis. For the matrigel interface experiment, the gel was not completely cured because the matrigel was only incubated for 30 min. The chip was tilted at 30°-45° for 5–10 min with two chambers filled with RPMI 1640 medium. During this time, the soft matrigel gradually formed the funnel-like cavity (matrigel I) due to gravity. Following that, the medium was absorbed completely. Next, 2 μl of matrigel was injected into the cavity, formed matrigel II and joined with matrigel I as a new entity. The cell loading procedures were the same as for the control group. Finally, the MDA-MB-231 cells attached to the side of the matrigel I before the device was incubated for further experiments. All experiments including the control experiments have been performed over five times.

## Conclusions

Metastatic MDA-MB-231 breast cancer cells exhibit strikingly different invasive behaviors in homogenous matrigel and heterogeneous matrigel with interior interfaces. The latter was designed to simulate the heterogeneous ECM environment *in vivo*. The experiments demonstrated that MDA-MB-231 cells failed to invade a homogenous 100% matrigel. However, in the matrigel with an interior interface, the cells detected and followed the localized heterogeneous structures and invaded the gel. Interestingly, the interface guided cell invasion at early stages, and the cells exhibited strong collective invasive behaviors later, such as finger-like invasion. Following corresponding theoretical simulation and analysis, we argue that this phenomenon is due to the combined effects of the matrigel interface structures, the cell-cell communication and the guidance of an FBS growth factor gradient. The cell invasion microenvironment is a heterogeneous landscape that greatly affects cell invasive behavior and the overall cell invasion patterns. Future experiments will focus on developing ECM in a variety type with heterogeneous gel density, pore sizes of the ECM and studying the corresponding cell collective invasion. Chemotherapy gradients will also be applied to target cell behaviors and drug resistance in time and space.
